# Effects of Mobile App-Based Mindfulness Practice on Healthcare Workers: a Randomized Active Controlled Trial

**DOI:** 10.1007/s12671-022-01975-8

**Published:** 2022-09-16

**Authors:** Shian-Ling Keng, Joseph Wei Ern Chin, Maleyka Mammadova, Irene Teo

**Affiliations:** 1 Department of Psychology, Jeffrey Cheah School of Medicine and Health Sciences, Monash University Malaysia, Subang Jaya, Malaysia; 2 Yale-NUS College, Singapore, Singapore; 3 Center for Sleep and Cognition, NUS Yong Loo Lin School of Medicine, Singapore, Singapore; 4 Center for Population Health Sciences, Lee Kong Chian School of Medicine, Nanyang Technological University, Singapore, Singapore; 5 Duke-NUS Medical School, Programme in Health Services and Systems Research, Singapore, Singapore

**Keywords:** Mindfulness, Mobile app interventions, COVID-19, Healthcare workers, Psychological health

## Abstract

**Objectives:**

Amidst the COVID-19 pandemic, healthcare workers (HCWs) may be at greater risk of suffering from psychological distress compared to the general population. This study aimed to investigate the effects of mindfulness practice as delivered using *Headspace* on psychological and cognitive outcomes among HCWs in Singapore.

**Methods:**

A total of 80 HCWs were recruited and randomly assigned to engage in either 3 weeks (10 min/day) of mindfulness practice using *Headspace* or an active control condition (*Lumosity*; involving playing cognitive games). Participants were administered several self-report measures and two working memory (digit span) tasks at pre- and post-intervention, and one-month follow-up.

**Results:**

There were no significant between-condition changes on any outcome variables from pre- to post-intervention. From pre-intervention to 1-month follow-up, there were significantly greater improvements among *Headspace* participants on fear of COVID-19 (*p* = .005), compassion satisfaction (*p* = .007), trait mindfulness (*p* = .002), self-compassion (*p* = .005), sleep quality (*p* = .002), and the forward digit span task (*p* < .001). Several outcomes were mediated by increases in trait mindfulness or self-compassion.

**Conclusions:**

Use of *Headspace* may lead to downstream benefits in reducing distress and improving psychological health outcomes among HCWs. The findings have implications for improving psychological support resources for HCWs amidst a pandemic.

**Trial Registration:**

ClinicalTrials.gov (Identifier: NCT04936893).

The coronavirus disease 2019 (COVID-19) pandemic has resulted in adverse economic, social, and psychological consequences for many around the globe. A vulnerable population consists of healthcare workers (HCWs), who are at greater risk of experiencing depression, anxiety, stress, and worsened sleep quality due to concerns regarding one’s health risk (including the risk of transmitting the virus to one’s family members) and increased workload (Cai et al., 2020; Ferini-Strambi et al., 2020; Lai et al., 2020; Liang et al., 2020; Shechter et al., 2020). A meta-analysis involving HCWs from mainly China found that at least one in five HCWs showed symptoms of depression and anxiety, and four in ten reported having difficulties with sleep or insomnia during the pandemic (Pappa et al., 2020). Additionally, HCWs have been found to be consistently at high risk of experiencing posttraumatic stress disorder (PTSD) symptoms in three major pandemic outbreaks in the past two decades (Carmassi et al., 2020). These findings highlight the deleterious impact of the COVID-19 pandemic on HCWs’ psychological well-being.

Past research has documented various negative effects of compromised psychological health among HCWs. Del Campo et al. (2017) found that HCWs with pre-existing depression or anxiety were at greater risk of developing musculoskeletal disorders than those without, even after adjusting for exposure risk factors in their occupations. Poor sleep has been linked with deterioration of executive and concentration-related procedures, as well as lowered attentional performance among HCWs (Di Muzio et al., 2020; Tempesta et al., 2013; Vinstrup et al., 2020). Not surprisingly, high levels of depression, anxiety, and stress have also been associated with poorer cognitive functioning, including compromised memory performance among nurses (Maharaj et al., 2019). HCWs are also found to be at increased risk of burnout. For example, 43.5% of HCWs working in an oncology setting in Singapore reported significant symptoms of burnout during an early lockdown period in 2020 (Ng et al., 2020). Burnout has also been associated with higher levels of compassion fatigue among HCWs, which negatively affects patient care (Kase et al., 2022).

Considering the negative psychological effects of the COVID-19 pandemic on HCWs, it is pertinent to examine effective strategies to mitigate negative psychological symptoms experienced by HCWs. Among various stress reduction strategies, mindfulness training is a promising strategy to help alleviate psychological distress and increase resilience among HCWs, given its extensive evidence base as a psychological intervention for improving mental health outcomes (Blanck et al., 2018; Keng et al., 2011; Wasson et al., 2020). In the context of the COVID-19 pandemic, there is a critical need to develop brief interventions or self-help practices to support HCWs in coping with stress, given the time demanding nature of their work.

Much research to date has demonstrated the benefits of mindfulness-based interventions for HCWs. For example, mindfulness-based stress reduction (MBSR; Kabat-Zinn, 1990), a group-based mindfulness training program, has been found to be effective at reducing perceived stress and burnout, and in improving mental and physical well-being, self-compassion, and satisfaction with life among HCWs (Goodman & Schorling, 2012; Shapiro et al., 2005). Despite the efficacy of MBSR, its relatively extensive time commitment (8 weekly sessions of 2.5 h each, and 30 to 40 min of daily practice) creates challenges for HCWs with a busy work schedule to commit to attending the program (Dobkin et al., 2012). Further, many HCWs are subject to shift work hours, which presents another barrier to participation in more intensive and regular-time interventions. More recent work has begun to examine whether brief mindfulness-based practices delivered using mobile applications may be an accessible and effective avenue for HCWs to practice and benefit from mindfulness training. Mobile-based mindfulness applications usually contain audio-recorded, guided meditations such as body scan, mindful breathing, and mindful walking, and users can access these practices using their smartphone device at their own convenience (Plaza et al., 2013). In a review of 560 existing mindfulness-based mobile applications available in the market, Mani et al. (2015) identified *Headspace* as an application that scores highly across several domains such as engagement, functionality, visual esthetics, information quality, and subjective quality.

Several studies have demonstrated that delivery of mindfulness training using a mobile application is associated with improvements in psychological health for HCWs. Wen et al. (2017) examined the effects of self-guided usage of *Headspace* for 4 weeks in a sample of medical staff, and found a significant increase in trait mindfulness from pre- to post-intervention. Another study by Zollars et al. (2019) also found significant increases in mindfulness and mental well-being, as well as decreases in perceived stress following 4 weeks of daily usage of *Headspace*. These studies however did not include a control group, which limits the inference of causal effects of the app. In a randomized controlled study by Yang et al. (2018), use of *Headspace* was found to be more effective than a waitlist control condition in improving well-being and observing (a facet of trait mindfulness), as well as reducing perceived stress in a sample of medical students. As the majority of studies on mindfulness for HCWs have focused on self-reported mental health outcomes, it is unknown whether app-guided mindfulness practice may also have an impact on specific domains of cognitive performance, such as working memory, which is known to deteriorate under conditions of high stress (Brand et al., 2000).

Existing studies have also compared the effects of mobile app-based mindfulness practice with those of in-person mindfulness classes. Wylde et al. (2017) found that self-guided usage (at least once a week, for 4 weeks) of *Headspace* led to significantly greater ability to act with awareness (a facet of trait mindfulness) compared to a traditional in-person mindfulness intervention in a sample of novice nurses. The authors noted that the improvement observed in the *Headspace* condition could be due to increased frequency of practices as a result of greater accessibility to self-guided practices as compared to live, guided instructions by an instructor. In Orosa-Duarte et al. (2021), engagement in mobile app-based mindfulness practices was associated with significant reductions in trait anxiety compared to a no-intervention control condition. Relative to the control condition, participants in both app-based and in-person interventions demonstrated significant increases in mindfulness and self-compassion. Taken together, these studies suggest that mobile app-guided mindfulness practice may be as effective as in-person mindfulness training in improving self-reported psychological outcomes in the HCW population.

Despite these promising findings, little work to date has evaluated the effects of mobile-guided mindfulness practice as a tool to buffer against psychological symptoms and improve cognitive performance in the context of an ongoing global pandemic. Mindfulness practice may be particularly beneficial for HCWs as a stress reduction tool as it can serve to enhance HCWs’ awareness of their moment-to-moment experiences without being overwhelmed by them. The latter can be particularly challenging as HCWs face increased workload and attentional demands, and are at greater vulnerability to anxiety and stress due to the nature of COVID-19 as a highly contagious disease. Mindfulness practice may enable HCWs to focus their attention on their tasks at hand more effectively while remaining in touch with their inner experiences, which is crucial for effective delivery of patient care (Braun et al., 2019). Mindfulness practice also promotes decentering, a process involving de-identifying from one’s negative thoughts and emotions and being able to view them as passing mental events (Sauer & Baer, 2010). This may reduce rumination and avoidance, which are known to be cognitive processes underlying depression and anxiety (McLaughlin & Nolen-Hoeksema, 2011; Spinhoven et al., 2014). Mindfulness practice may also lead to increases in self-compassion (Shapiro et al., 2005), as the practice promotes an accepting and non-judgmental attitude in relating to one’s experiences.

Additionally, mindfulness practice may lead to improvements in professional quality of life among HCWs. A systematic review (Luken & Sammons, 2016) showed that mindfulness training is effective in reducing job burnout among HCWs and teachers, though the review did not focus on training delivered using mobile app platforms. In a study involving hospital chaplains, mindfulness, self-compassion, and a supportive structure were each found to be protective factors against burnout (Hotchkiss & Lesher, 2018). By reducing burnout, mindfulness training may enable HCWs to maintain a higher level of compassion satisfaction at work. It remains to be examined whether briefer forms of mindfulness practice as delivered using a mobile app may exert an effect on professional quality of life among HCWs.

The current study aimed to investigate the effects of 3 weeks of brief mindfulness practice delivered using a mobile application, *Headspace*, on a range of psychological and cognitive health outcomes in a sample of HCWs based in Singapore. We hypothesized that compared to an active control condition (*Lumosity*), participants in the mindfulness condition (*Headspace*) would report greater improvements in psychological symptoms (depression, anxiety, PTSD symptoms, and fear of COVID-19), personal well-being, professional quality of life (burnout and compassion satisfaction), sleep quality, and short-term memory from pre- to post-intervention, and from pre-intervention to one-month follow-up. We further hypothesized that changes in trait mindfulness and self-compassion would mediate the effects of *Headspace* on these outcomes at post-intervention and 1-month follow-up. It was also predicted that there would be a positive association between duration of mindfulness practice and improvements in the outcome measures.

## Methods

### Participants

A total of eighty HCWs participated in the study. Participants were recruited through a research participant database hosted by Duke-NUS medical school, word of mouth, and social media. The recruitment poster highlighted that the goal of the study was to evaluate the effects of a stress management mobile app among health care workers during the COVID-19 pandemic, without mentioning mindfulness training or *Headspace*. Inclusion criteria were as follows: a HCW based in Singapore, aged between 21 and 60, proficient in English, and owning a smartphone (iOS or Android) with Wi-Fi or data access. Participants were excluded if they were engaging in regular mindfulness practice, defined by practicing a minimum of two to three times a week for 10 to 15 min each time within the past six months. An a priori power analysis indicated that the sample size needed for the study was 81, assuming an alpha level of 0.05, a small-to-moderate effect size of *f*^2^ = 0.10, and 80% power. The estimated effect size was based on findings from a meta-analysis on the effects of online mindfulness-based interventions (Spijkerman et al., 2016). Each participant was paid 50 Singapore dollars (equivalent to approx. $38USD) upon completing all study assessments. The study was approved by the National University of Singapore’s Institutional Review Board (NUS-IRB-2020–51).

Table 1 presents the demographic characteristics of participants across both experimental conditions (*n* = 80). The sample’s mean age was 30.18 (SD = 6.19) years, with a range of 22 to 54 years. A majority of participants were of Chinese ethnicity (*n* = 64; 80%) and female (*n* = 72; 90%). Most of the participants attained an undergraduate degree or above (*n* = 68; 85%). More than half the sample were nurses (*n* = 47; 58.75%) and worked on a shift schedule (*n* = 46; 57.5%). Finally, approximately half the sample reported having exposure to COVID-19 patients or suspected cases (*n* = 41; 51.25%), and prior experience with (but not regularly practising) mindfulness (*n* = 39; 48.8%). One participant in the *Headspace* condition withdrew from the study upon completing the baseline assessment, due to not being able to commit to daily mindfulness practice.

**Table 1:** Sample characteristics

Demographic variable	Headspace (*n* = 40)		Lumosity (*n* = 40)		Full sample (*n* = 80)	
	*n*	%	*n*	%	*n*	%
Gender						
Female	36	90	36	90	72	90
Male	4	10	4	10	8	10
Marital status						
Single	21	52.5	23	57.5	44	55
Married	14	35	8	20	22	27.5
In a relationship	5	12.5	6	15	11	13.75
Divorced/separated	0	0	2	5	2	2
Widowed	0	0	1	2.5	1	1.25
Ethnicity						
Chinese	34	85	30	75	64	80
Malay	2	5	3	7.5	5	6.25
Indian	2	5	3	7.5	5	6.25
Others	2	5	4	10	6	7.5
Highest education						
Bachelor’s degree	31	77.5	26	65	57	71.25
Graduate degree	5	12.5	6	15	11	13.75
Diploma	2	5	7	17.5	9	11.25
Current student	2	5	1	2.5	3	3.75
Previous experience with mindfulness						
Yes	21	52.5	18	45	39	48.8
Exposure to COVID-19 patients and suspected patients						
Yes	20	50	21	52.5	41	51.2
Shift schedule						
Yes	21	52.5	25	62.5	46	57.5

Following guidelines used by Hooper et al. (2010), at baseline, 77.5% of HCWs met the minimum cutoff score (≥ 23) for burnout (*n* = 62) as defined by the Professional Quality of Life Scale (ProQOL). On the depression subscale of DASS-21, more than half of the sample (*n* = 50; 62.5%) scored in the normal range of depressive symptoms, with 27.5% scoring in the mild to moderate range, and 5% scoring in the severe range. Similarly, more than half of the sample (*n* = 50; 62.5%) scored in the normal range of anxiety, followed by 32.5% scoring mild to moderate range, and 10% scoring in the severe to extremely severe range of anxiety.

### Procedure

**Fig. 1:**
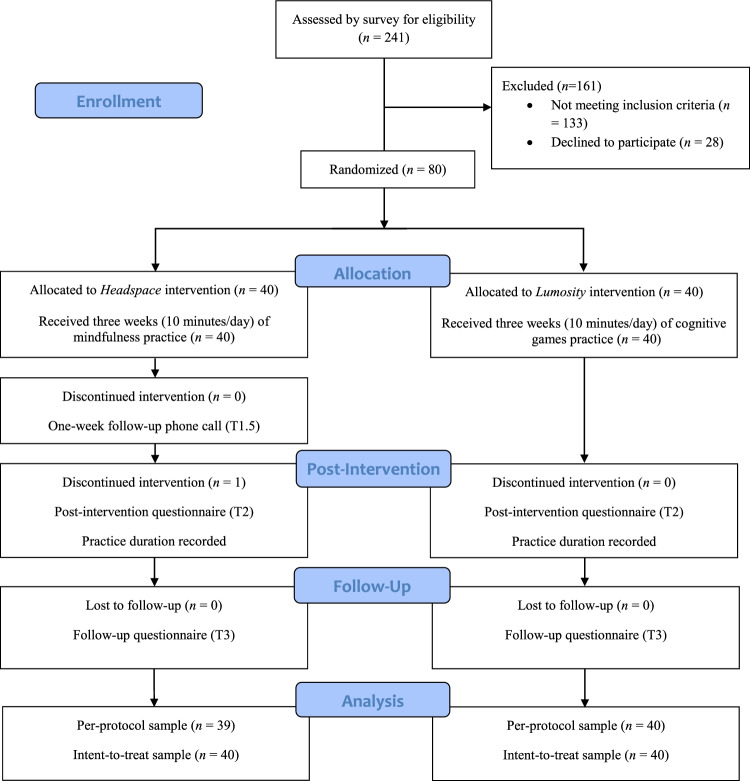
Study procedure

Potential participants were directed to an online pre-screening survey prior to enrolling in the study (see Fig. 1). The pre-screening survey consisted of several questions assessing participants’ eligibility to enrol in the study. Eligible participants were invited to schedule an individual assessment session (T1) conducted online with an experimenter. In this session, participants were briefed about the study and provided informed consent to participate in the study. Participants then completed a battery of baseline measures, including two digit span tests (see Measures and Tasks section below). They were then randomly assigned via block randomization (http://www.randomizer.org) to either the experimental condition (*Headspace*) or the control condition (*Lumosity*). Each participant then received an orientation to their assigned mobile app*.* The orientation included a short introduction to mindfulness practice or cognitive training, a guide to using the assigned app, and a short, 10-min practice exercise using the app platform. Both conditions were matched on the length and modality used during orientation training.

*Headspace* participants were instructed to adhere to *Headspace*’s 10-day basic course, by completing one 10-min practice each day according to the sequence of practices outlined in the course (i.e., day 1 through day 10) before moving on to other mindfulness practices within the app at their choice. Examples of practices included in the 10-day course were mindful breathing, mindfulness of thoughts, and mindfulness of sounds. *Lumosity* participants were told to complete the *Daily Training* component (consisting of three free daily games, involving problem solving, memory, and attention, averaging 10 min in total) or 10 min’s worth of games within the app every day. Participants were instructed to engage in daily practice using their assigned app for 3 weeks. At the end of T1 session, the experimenter scheduled a 1-week follow-up phone coaching call with *Headspace* participants. Considering recent research highlighting potential adverse effects associated with mindfulness practice (Britton, 2019), the phone coaching call was conducted to address any questions or challenges that might arise during participants’ engagement with mindfulness practice. Each call lasted an average of 5 to 10 min.

At T2 session, which was conducted following completion of 21 days of daily app practice, all participants completed the same battery of outcome measures and performance tasks, with the addition of a few questions assessing experimenter credibility and enthusiasm. At the end of the session, the experimenter recorded the total duration of mindfulness practice completed by *Headspace* participants by checking on individual participants’ apps (as this data was recorded automatically by the app). One month following T2 session, participants attended an individual follow-up assessment session (T3) and completed the same battery of outcome measures. In this session, participants in the *Lumosity* condition were also given information about mindfulness practice and *Headspace*. All participants were debriefed about the study and given a list of psychological resources.

### Measures

#### Demographic Data Form

A demographic data form was administered to gather information pertaining to participants’ gender, age, ethnicity, marital status, highest education, workplace position, profession, and hours spent at work weekly. Additionally, participants were asked if they were dealing with COVID-19 patients or patients suspected to be infected in their workplace, alongside an approximate number of COVID-19 patients they encountered weekly. The form also requested for information regarding history of receiving psychotherapy and psychiatric medications, and previous experience with mindfulness practice.

#### Depression, Anxiety, and Stress Scales-21

The Depression and Anxiety subscales of the Depression, Anxiety, and Stress Scales-21 (Lovibond & Lovibond, 1995) were administered to assess symptoms of depression and anxiety within the past week. Participants were instructed to rate statements (e.g., “I couldn’t seem to experience any personal feelings at all”) based on how much each item applied to them on a scale from 0 to 3 (0 = *Did not apply to me at* all to 3 = *Applied to me very much or most of the time*). Scores are totalled and multiplied by 2 to derive a subscale score. The scale demonstrated good internal reliability on subscales of depression (α = 0.88) and anxiety (α = 0.82) in a sample of adults from the UK (Henry & Crawford, 2005). In this sample, the internal consistency (as measured by Cronbach’s alpha) and true reliability (as measured by McDonald’s omega) for the depression and anxiety subscales at T1 were good (α = 0.86; McDonald’s ω = 0.86) and acceptable (α = 0.78; McDonald’s ω = 0.78) respectively.

#### Fear of COVID-19 Scale

The Fear of COVID-19 Scale (Ahorsu et al., 2022) is a 7-item scale assessing one’s anxiety and fear about COVID-19. Sample statements on the scale include “I am most afraid of coronavirus-19” and “My heart races or palpitates when I think about getting coronavirus-19.” Participants rated these statements on a 5-point scale (1 = *Strongly disagree;* 5 = *Strongly agree*). The scale demonstrated good internal consistency (*α* = 0.82) and acceptable test–retest reliability (ICC = 0.72) in a sample of Iranian participants (Ahorsu et al., 2022). In this sample, the scale demonstrated good internal constancy and true reliability (at T1: α = 0.86; McDonald’s ω = 0.86).

#### Posttraumatic Stress Disorder CheckList–Civilian Version

Posttraumatic Stress Disorder CheckList–Civilian Version (PCL-C; Weathers et al., 1993) is a 17-item self-report measure of PTSD symptoms. Participants were asked to rate the extent to which they experienced various PTSD symptoms (e.g., “repeated, disturbing memories, thoughts, or images of a stressful experience from the past”) in the past month. Items were rated on a 5-point Likert-type scale (1 = *Not at all;* 5 = *Extremely*). The PCL-C has demonstrated excellent internal consistency (*α* = 0.94) in a sample of motor vehicle accident victims and sexual assault victims (Blanchard et al., 1996). In this study, the checklist’s internal consistency and true reliability were excellent (at T1: α = 0.91; McDonald’s ω = 0.91).

#### Personal Well-being Index

The Personal Wellbeing Index (International Wellbeing Group, 2013) is a 9-item self-report questionnaire measuring multiple domains of subjective well-being (SWB) such as general life satisfaction, personal health, relationships, safety, and community connectedness. Participants were asked to rate how satisfied they feel on each item on a scale from 1 to 10 (1 = *No satisfaction at all* to 10 = *Completely Satisfied*). Items are combined to yield an average score for SWB. The scale displayed good internal consistencies (*α* = 0.73 to 0.80) among individuals from Australia and Hong Kong (Lau et al., 2005). In this study, the scale’s internal consistency and true reliability at T1 were good (α = 0.88; McDonald’s ω = 0.89).

#### Professional Quality of Life Scale

Burnout and compassion satisfaction were assessed using two subscales of the ProQOL (Stamm, 2010), which assesses how one feels in relation to one’s professional work as a helper. In this study, the term “helper” was modified to “healthcare worker” to suit the study’s sample. Each subscale contains ten statements, and participants rated these statements on a 5-point scale (1 = *Never* to 5 = *Very often*). Sample items include “I get satisfaction from being able to help people” (compassion satisfaction), and “I feel worn out because of my work as a healthcare worker” (burnout). At T1, the subscales’ Cronbach’s alphas were 0.77 (McDonald’s ω = 0.77) and 0.88 (McDonald’s ω = 0.88) for burnout and compassion satisfaction respectively.

#### Perceived Sleep Quality

Perceived sleep quality was measured using a one-item scale derived from the Pittsburgh Sleep Quality Index (Buysse et al., 1989), which assesses one’s self-reported quality of sleep over the past month. The item “During the past month, how would you rate your sleep quality overall?” was rated on a 4-point scale (1 = *Very bad* to 4 = *Very good*).

#### Five Facet Mindfulness Questionnaire

The Five Facet Mindfulness Questionnaire (FFMQ; Baer et al., 2006) is a 39-item measure assessing individual differences in trait mindfulness. The measure assesses five mindfulness skills, namely observing, describing, acting with awareness, non-judging of inner experience, and non-reactivity to inner experience. Sample items include “I pay attention to physical experiences, such as the wind in my hair or sun on my face” and “I perceive my feelings and emotions without having to react to them.” The subscales demonstrated good to excellent internal consistencies (α = 0.75 to 0.91) in a sample of undergraduate students (Baer et al., 2006). In the original scale, participants are required to rate statements on a 5-point scale (1 = *Never or very rarely true* to 5 = *Very often or always true*). Due to an administrative error on the survey platform, the current study’s FFMQ was administered using a 4-point (1 = *Never or very rarely true* to 4 = *Often true*) instead of 5-point scale. Despite this error, the overall scale demonstrated good internal consistency (at T1: α = 0.88) in this sample. McDonald’s ω could not be estimated for FFMQ due to an error related to negative or zero item covariances. Therefore, for this measure, we reported only its Cronbach’s alpha.

#### Self-Compassion Scale

The Self-Compassion Scale (SCS; Neff, 2003) is a self-report scale assessing the tendency to be kind towards oneself. The SCS consists of the following subscales: self-kindness, self-judgment, common humanity, isolation, mindfulness, and over-identification. Respondents are required to rate 26 statements on a 5-point scale (1 = *Almost never* to 5 = *Almost always*). Items on the scale include “I try to be loving towards myself when I’m feeling emotional pain” and “I try to see my failings as part of the human condition.” The measure demonstrated good test–retest reliability and excellent internal consistency (α = 0.92) in a sample of undergraduate students (Neff, 2003). In this study, the scale demonstrated excellent internal consistency and true reliability (at TI: α = 0.93; McDonald’s ω = 0.92).

#### Digit Span Tests–Forward and Backward

Forward and backward Digit Span Tests (DST-F/B) of the Wechsler Adult Intelligence Scale (Wechsler, 2008) were administered to assess working memory. There are seven ascending digit spans for both forward (3 to 9 digits) and backward (2 to 8 digits), with two trials in each digit span. The length of the digit span increases by one digit if the participant answers at least one of the two trials within that digit span correctly. However, both trials of each digit-span length are administered to the participant even if the participant answers the trial correctly. If a participant fails to answer both trials of any digit span, the test ends. Scoring is based on the number of trials the participant answers correctly, yielding a range of 0–14 points on both the forward and the backward DSTs. The tests were administered online following recommendations by Pearson (2021).

### Data Analyses

Statistical Package for Social Sciences Version 26 was used to conduct data analyses. Data were cleaned and checked for outliers and distribution (e.g., normality) prior to analyses. Participants across conditions were compared on their baseline characteristics using chi-square tests for categorical variables, and independent samples *t*-tests for continuous variables. Next, a series of hierarchical regressions were conducted to identify potential covariates to be included in the primary analyses. Potential covariates (age, gender, education level, ethnicity, marital status, hours worked/day, exposure to COVID-19 cases, past experience with mindfulness practice, experimenter credibility, and enthusiasm) were regressed individually on T2 scores of each outcome variable, controlling for the respective T1 score. Variables that emerged as significant predictors of T2 scores were included as covariates in the subsequent primary analyses.

A series of hierarchical regressions were conducted on the per-protocol sample (*n* = 79) to examine the effects of experimental condition on T2 scores, controlling for T1 scores and any identified covariates. The same analyses were repeated using T3 scores as DVs. Considering the multiple dependent variables, only results with *p* < 0.01 were marked as significant. These analyses were also carried out using the intent-to-treat (ITT) sample (*n* = 80). For the ITT sample, missing data from one participant who withdrew from the study after the baseline assessment was replaced using the last-observation-carried-forward approach.

For outcomes with a significant effect of experimental condition, mediational analyses were conducted using the PROCESS Macro (Hayes, 2017; model 4), with change in mindfulness being tested as a mediator of the effects of experimental condition on the outcome variables. The same analyses were repeated with self-compassion as the mediator. For participants in the *Headspace* condition, correlational analyses were conducted to examine the association between practice duration and changes on each of the outcome measures from T1 to T2, and from T1 to T3.

## Results

### Randomization Check

A series of chi-square tests displayed no significant baseline differences between conditions on any of the categorical variables, including gender, marital status, ethnicity, education, prior experience with mindfulness practice, exposure to COVID-19 cases/suspected cases, and shift status, *p*s > 0.26. Independent samples *t*-tests revealed that participants did not differ across condition on their ratings of experimenter credibility and enthusiasm, and on any of the outcome measures at baseline, *p*s > 0.05.

### Covariate Analyses

From time 1 to time 2, increases in anxiety were positively associated with education level (*p* = 0.02) and increases in secondary traumatic stress were associated with working on a shift schedule (*p* = 0.009). Further, increases in fear of COVID-19 were associated with gender (being female; *p* = 0.045) and increases in sleep quality were positively associated with age (*p* = 0.009). Decreases in DST-B were associated with exposure to COVID-19 cases (*p* = 0.005). From time 1 to time 3, increases in personal well-being were associated with shift status (*p* = 0.02), and decreases in PTSD symptoms were related with ethnicity (being Chinese; *p* = 0.037). These variables were entered as covariates in the primary analyses pertaining to the respective DVs.

### Effects of Headspace from Pre- to Post-intervention

Table 2 presents the descriptive and test statistics for the effects of experimental condition on changes in each outcome variable from pre- to post-intervention. There were no significant between-group differences on changes in any of the outcome variables, *p*s > 0.05. These results were replicated when the analyses were conducted using the ITT sample.

**Table 2:** Descriptive and test statistics for the effects of headspace vs. lumosity from time 1 to time 2 (*n* = 79)

Outcome	Headspace		Lumosity		Group effects		
	*Time 1*	*Time 2*	*Time 1*	*Time* 2	β	*p*	*f*^2^
Depression	6.97 (7.51)	5.10 (5.05)	8.05 (6.05)	7.78 (6.98)	− 0.166	0.057	0.03
Anxiety	5.72 (5.97)	4.44 (4.68)	6.78 (5.6)	6.25 (5.75)	− 0.126	0.148	0.03
Fear of COVID-19	13.77 (5.47)	11.64 (4.50)	13.38 (4.33)	11.75 (4.30)	− 0.046	0.514	0.01
PTSD symptoms	30.36 (9.91)	27.90 (7.63)	31.83 (9.73)	30.08 (8.72)	− 0.092	0.336	0.01
Personal well-being	5.87 (1.10)	7.18 (0.96)	6.02 (0.95)	7.03 (1.29)	0.137	0.107	0.03
Compassion satisfaction	38.28 (5.89)	38 (6.26)	37.98 (5.90)	37.15 (5.54)	0.053	0.483	0.01
Burnout	26.28 (5.07)	24.97 (5.40)	26.05 (5.46)	25.63 (5.49)	− 0.077	0.308	0.01
Perceived sleep quality	2.74 (0.60)	2.87 (0.52)	2.92 (0.69)	2.75 (0.84)	0.094	0.359	0.01
Trait mindfulness	103.49 (13.96)	107.15 (12.68)	99.48 (12.73)	101 (10.34)	0.16	0.054	0.04
Self-compassion	3.06 (0.67)	3.20 (0.64)	3.00 (0.59)	3.08 (0.51)	0.063	0.360	0.01
Digit-span forward	8.54 (2.63)	9.95 (2.15)	9.00 (2.50)	10 (2.27)	0.037	0.707	0.003
Digit-span backward	8.43 (2.46)	9.72 (2.82)	8.23 (2.39)	9.68 (2.62)	0.015	0.874	< 0.001

Test statistics are for the second step of hierarchical multiple regression equations predicting time 2 scores, with time 1 scores and covariates entered at the first step, and group assignment entered at the second step

### Effects of Headspace from Pre-intervention to One-Month Follow-up

Compared to participants assigned to *Lumosity*, *Headspace* participants reported significantly greater decreases in fear of COVID-19, and greater increases in compassion satisfaction, sleep quality, trait mindfulness, self-compassion, and DSF scores from pre-intervention to 1-month follow-up (see Table 3). Effect sizes for significant outcomes ranged between small and medium. No significant between-group differences were found on changes in depression, anxiety, burnout, PTSD symptoms, well-being, secondary traumatic stress, or DST-B scores, *p*s > 0.02. ITT analyses yielded similar results.

**Table 3:** Descriptive and test statistics for the effects of headspace vs. lumosity from time 1 to time 3 (*n* = 79)

Measure	Headspace		Lumosity		Group effects		
	Time 1	Time 3	Time 1	Time 3	β	*p*	*f*^2^
Depression	6.97 (7.51)	4.31 (4.32)	8.05 (6.05)	7.95 (8.50)	− 0.222	0.021	0.07
Anxiety	5.72 (5.97)	4.46 (4.39)	6.78 (5.60)	5.43 (5.82)	− 0.046	0.638	0.003
Fear of COVID-19	13.77 (5.47)	10.13 (3.70)	13.38 (4.33)	12.65 (4.95)	− 0.239	0.005	0.11
PTSD symptoms	30.36 (9.91)	24.33 (5.74)	31.83 (9.73)	27.73 (9.27)	− 0.153	0.079	0.04
Personal well-being	5.87 (1.10)	8.46 (0.88)	6.02 (0.95)	8.20 (1.21)	0.193	0.022	0.06
Compassion satisfaction	38.28 (5.89)	39.44 (6.15)	37.98 (5.90)	36.90 (6.18)	0.183	0.007	0.10
Burnout	26.28 (5.07)	24.44 (4.78)	26.05 (5.46)	25.58 (5.56)	− 0.127	0.098	0.02
Perceived sleep quality	2.74 (0.60)	4.00 (0.69)	2.92 (0.69)	3.60 (1.06)	0.298	0.002	0.12
Trait mindfulness	103.49 (13.96)	109.03 (14.65)	99.48 (12.73)	99.30 (11.52)	0.255	0.002	0.12
Self-compassion	3.06 (0.67)	3.38 (0.55)	3.00 (0.59)	3.08 (0.54)	0.208	0.005	0.10
Digit-span forward	8.54 (2.63)	10.62 (2.09)	9.00 (2.50)	8.90 (3.23)	0.356	< 0.001	0.19
Digit-span backward	8.43 (2.46)	10.18 (2.51)	8.23 (2.39)	9.38 (2.35)	0.182	0.079	0.04

Test statistics are for the second step of hierarchical multiple regression equations predicting time 3 scores, with time 1 scores and covariates entered at the first step, and group assignment entered at the second step

### Trait Mindfulness as a Mediator of Intervention Effects

As there were no between-condition differences on any of the outcome variables from T1 to T2, mediational analyses focused on outcome variables with a significant effect of experimental condition from T1 to T3. From T1 to T3, changes in trait mindfulness significantly mediated the effects of *Headspace* on changes in compassion satisfaction (indirect = 1.17, *SE* = 0.67, 95% CI [0.11, 2.70]) and burnout (indirect =  − 1.21, *SE* = 0.68, 95% CI [− 2.80, − 0.14]). There was no significant indirect effect of trait mindfulness on changes on any of the other outcome variables. The mediational results were replicated in the ITT sample.

### Self-compassion as a Mediator of Intervention Effects

From T1 to T3, there was a significant indirect effect of changes in self-compassion on depressive symptoms (indirect =  − 1.20, *SE* = 0.61, 95% CI [− 2.61, − 0.23]) and PTSD symptoms (indirect =  − 1.18, *SE* = 0.69, 95% CI [− 2.71, − 0.07]). Self-compassion also mediated the effects of experimental condition on changes in compassion satisfaction (indirect = 1.10, *SE* = 0.61, 95% CI [0.10, 2.51]) and burnout (indirect =  − 1.52, *SE* = 0.71, 95% CI [− 3.09, − 0.30]). There was no significant mediation by self-compassion on any of the other outcome variables. Analyses using the ITT sample yielded similar results overall.

### Association Between Practice Duration and Changes in Outcome Measures

On average, participants in the *Headspace* condition completed 185.31 min (SD = 88.65) of mindfulness practice by T2 (3 weeks after baseline) and 307.64 min (SD = 209.39) at T3. Relative to the amount of practice stipulated based on the study protocol (10 min per day × 21 days = 210 min), the average duration of practice recorded approximates 88.2% of completion rate. More specifically, more than half of the participants (*n* = 22; 56.41%) completed at least 80% of the required practice duration, whereas a third of the participants (*n* = 12; 30.76%) completed between 40 and 80% of the required practice duration. Approximately 12.8% of participants (*n* = 5) completed between 20 and 40% of the required practice duration.

Table 4 presents results from a series of correlational analyses examining the association between mindfulness practice duration and changes in outcome measures for participants in the *Headspace* condition. There were no significant associations between practice duration and changes in any of the outcome variables at T2 and T3, *p*s > 0.02.

**Table 4:** Association between mindfulness practice duration and changes in outcome measures (*n* = 39)

Variable	Mindfulness practice duration	
	Time 1 to time 2	Time 1 to time 3
Depression	− 0.06	− 0.07
Anxiety	0.16	0.17
Fear of COVID-19	0.06	− 0.03
PTSD symptoms	− 0.18	0.11
Personal well-being	0.02	− 0.08
Compassion satisfaction	0.11	0.06
Burnout	− 0.11	0.07
Perceived sleep quality	0.11	0.01
Trait mindfulness	0.18	0.18
Self-compassion	0.39*	0.18
Digit-span forward	− 0.17	0.10
Digit-span backward	− 0.08	− 0.31

^*^p < .05

## Discussion

The present study found that 3 weeks of mindfulness practice as administered using *Headspace* yielded benefits that are detected at 1-month follow-up. Immediately after the 3-week practice period, there were no between-condition differences on changes in any of the outcome measures. At 1-month follow-up, participants of *Headspace* demonstrated significantly greater improvements in fear of COVID-19, compassion satisfaction, sleep quality, trait mindfulness, self-compassion, and one of two short-term memory tasks. Several of these changes were mediated by improvements in trait mindfulness or self-compassion.

The finding that use of *Headspace* was not associated with differential improvements in any outcome measures from pre- to post-intervention was surprising, given that prior uncontrolled studies have demonstrated an association between use of mobile-app-based mindfulness practice and improvements in mental well-being among medical staff (Wen et al., 2017) and students (Zollars et al., 2019). The finding shows that in comparison to an active control group, use of *Headspace* does not confer an advantage in improving hypothesized outcomes. Within-group analyses (using paired sample *t* tests to compare pre- and post-intervention scores) however showed improvements in expected directions for the majority of assessed outcomes, namely trait mindfulness, self-compassion, depressive symptoms, anxiety, burnout, compassion fatigue, fear of COVID-19, working memory performance (both forward and backward versions of the DST), and well-being in the *Headspace* condition. It is plausible that a more intensive or longer intervention (i.e., longer periods of practice) may be required for mindfulness training to exert a differentiable impact on individuals’ trait mindfulness, self-compassion, and mental health. Notably, existing research demonstrating positive effects of mindfulness training on HCWs mostly employ in-person modes of training delivery (Duchemin et al., 2015; Fortney et al., 2013; Foureur et al., 2013), suggesting that in-person mindfulness training (with opportunities for live practice inquiry, modeling, and feedback) may be more effective in improving mental health outcomes, at least at the beginning stages of exposure to mindfulness practice.

At 1-month follow-up, several hypothesized benefits of *Headspace* began to emerge more clearly. Consistent with past research (Bergen-Cico et al., 2013; Richards & Martin, 2012), the study found improvements in trait mindfulness and self-compassion following use of *Headspace*, and demonstrated that these effects are not likely accountable for by expectancy or placebo. Mindfulness practice may enable HCWs to take a more decentered and non-judgmental perspective in relating to their thoughts and emotions, which may facilitate a kinder attitude in relating to themselves (Fissler et al., 2016). Interestingly, despite no between-condition differences in changes in anxiety, participants using *Headspace* also reported significantly greater reductions in fear of COVID-19 compared to control group participants. This suggests that brief mindfulness practice exerted a more pronounced effect in moderating perceived threat and fear that were more salient at the time of the study, when the COVID-19 pandemic was developing in Singapore, and much remained unknown regarding medical risks of COVID-19 and the capacity of the healthcare system in managing the pandemic.

At follow-up, participants using *Headspace* also reported greater improvements in compassion satisfaction and burnout compared to those in the control group. The findings are consistent with results from two uncontrolled studies demonstrating improvements in burnout and compassion satisfaction following participation in in-person mindfulness programs (Ceravolo & Raines, 2019; Hevezi, 2016). The results suggest that even brief periods of mindfulness practice could be effective in improving professional quality of life for HCWs, though the effects are more likely to emerge over a longer period of consistent practice. As articulated by Kabat-Zinn (2003), mindfulness practice is “akin to an art form that one develops over time” (p. 148), and regular practice is required for one to reap greater benefits from mindfulness training.

The finding that use of *Headspace* led to improvements in perceived sleep quality at follow-up corresponds with previous research showing a positive association between trait mindfulness and sleep quality among HCWs (Kemper et al., 2015; Liu et al., 2020). Within the larger literature, participation in established mindfulness-based interventions such as MBSR has been associated with improved sleep quality, though evidence is less consistent when MBSR is compared with active control conditions (Winbush et al., 2007). By utilizing an active control condition, this study provides evidence that brief mindfulness practice may result in improved sleep quality above and beyond expectancy or placebo effects. Notably though, sleep quality was assessed using a one-item measure derived from the PSQI, which may not capture all facets of sleep quality. Future studies should assess additional dimensions of sleep quality such as duration and sleep latency, and consider employing objective measures (e.g., polysomnography and actigraphy) to examine the effects of mindfulness on sleep.

The present study also provided evidence that regular mindfulness practice may lead to improvements in working memory, as demonstrated by significant time 1 to time 3 improvements on the forward version of the DST, compared to control group participants. The finding is consistent with studies finding improvements in working memory following exposure to intensive meditation retreat (Chambers et al., 2008) and several sessions of mindfulness training (Zeidan et al., 2010). Our study extended these findings by demonstrating these effects in a sample of HCWs working in a high stress context of dealing with the COVID-19 pandemic. Further, improvements in working memory are not likely attributable to the effects of general cognitive training, but rather forms of attentional training unique to mindfulness. Specifically, mindfulness training’s emphasis on cultivating sustained present moment awareness may increase individuals’ capacity to retain information in short-term memory.

The finding that changes in trait mindfulness and self-compassion mediated the effects of *Headspace* on selected outcomes at time 3 corresponds with established theoretical and empirical work positing the role of mindful awareness and self-compassion in explaining the effects of regular mindfulness practice (Baer, 2010; Duarte & Pinto-Gouveia, 2017; Keng et al., 2012). Interestingly, both trait mindfulness and self-compassion accounted for the effects of *Headspace* on burnout and compassion satisfaction, suggesting that shifts in these processes exert more impact on HCWs’ professional quality of life relative to other outcomes. An increase in the ability to attend to one’s experience mindfully and with kindness may lower burnout by disrupting maladaptive cognitive tendencies such as rumination (Heeren & Philippot, 2011) and promoting self-care (Slonim et al., 2015). These shifts may also facilitate a greater capacity to derive joy and satisfaction from helping others. Beyond burnout and compassion satisfaction, improvements in self-compassion also mediated the effects of *Headspace* on depression and PTSD symptoms at follow-up. The finding suggests that regular mindfulness practice helps facilitate a kinder and more accepting attitude towards oneself, which in turn reduces emotional reactivity and vulnerability to psychological symptoms.

The study found that mindfulness practice duration was not associated with changes in any outcome variables. It is plausible that relative to duration of practice, practice quality, or the quality of one’s attentional engagement during the practice could be a better predictor of psychological outcomes. In a randomized trial examining the effects of mindfulness training among smokers (Goldberg et al., 2014), changes in practice quality were found to predict changes in psychological functioning at posttreatment, when controlling for practice duration. Future research should explore whether practice quality would be a stronger mediator of the effects of mindfulness practice by administering validated measures such as the Practice Quality-Mindfulness (Del Re et al., 2013) scale.

Several strengths of the study include use of a randomized design with an active control condition, inclusion of a follow-up assessment, and objective tracking of practice duration. By implementing an active control condition, the study was able to demonstrate that the effects of *Headspace* above and beyond general expectancy or placebo effects. Furthermore, implementing a longitudinal design with three timepoints lends insight into longer-term effects of mindfulness practices through the application. Lastly, the study benefitted from using an objective measure (automatic tracking of practice duration using the app) to track duration of mindfulness practice in the *Headspace* condition.

### Limitations and Future Research

The study is not without its limitations. First, the study did not track practice duration for participants in the *Lumosity* condition, as the app did not have a feature of tracking the amount of practice over time. This precludes us from assessing the extent to which participants across both conditions were spending equivalent amounts of time using the apps. It is plausible that *Headspace* participants might have been more motivated to continue to use the app between time 2 and time 3 (if they perceived more benefit from mindfulness training versus those assigned to cognitive training), which may explain the advantage of *Headspace* versus *Lumosity* at time 3.

Further, use of self-report measures to assess psychological symptoms and emotional well-being is subject to recall and social desirability biases, as well as common method bias (i.e., bias arising from measuring multiple constructs using the same method). Future studies may look to implement diverse modes of assessments, such as psychophysiological assessments or interviews to mitigate the biases. It is also notable that participants in the *Headspace* condition received a brief phone coaching call from a research assistant 1 week into the intervention period, which might have served to increase their motivation to continue their mindfulness practice using the app. Future research should evaluate to what extent the benefits of using *Headspace* would sustain in the absence of guidance or coaching. On the other hand, it will be of value to examine whether a more intensive intervention (e.g., one that involves in-person mindfulness training combined with self-guided practice) would yield stronger intervention effects. Lastly, future work should examine whether there are individual traits (e.g., conscientiousness) that may moderate the efficacy of mobile app–based mindfulness practices, as well as investigate the extent to which mental health benefits of mindfulness training translate into improved patient care within the healthcare setting.

## Data Availability

All data are available at the Open Science Framework (https://osf.io/4v5q3/).
